# Nucleus Accumbens Response to Reward among Children with a Family History of Alcohol Use Problems: Convergent Findings from the ABCD Study^®^ and Michigan Longitudinal Study

**DOI:** 10.3390/brainsci12070913

**Published:** 2022-07-13

**Authors:** Meghan E. Martz, Jillian E. Hardee, Lora M. Cope, Katherine L. McCurry, Mary Soules, Robert A. Zucker, Mary M. Heitzeg

**Affiliations:** Department of Psychiatry and Addiction Center, University of Michigan, 4250 Plymouth Road, Ann Arbor, MI 48109, USA; jhardee@med.umich.edu (J.E.H.); lcope@med.umich.edu (L.M.C.); kathlmcc@med.umich.edu (K.L.M.); mfield@med.umich.edu (M.S.); zuckerra@med.umich.edu (R.A.Z.); mheitzeg@med.umich.edu (M.M.H.)

**Keywords:** fMRI, reward, nucleus accumbens, family history, alcohol, MID task

## Abstract

Having a family history of alcohol use problems (FH+) conveys risk for alcohol use in offspring. Reward-related brain functioning may play a role in this vulnerability. The present study investigated brain function in the nucleus accumbens (NAcc) associated with the anticipation of reward in youth with two biological parents with alcohol use problems (FH+2), one biological parent with alcohol use problems (FH+1), and no biological parents with alcohol use problems (FH-). Participants were from the large, national Adolescent Brain Cognitive Development (ABCD) Study (mean age: 9.93; 48% female; FH+2 n = 223, FH+1 n = 1447, FH- n = 9690) and the Michigan Longitudinal Study (MLS), consisting of community-recruited families with high rates of alcohol use disorder (mean age: 10.54; 39.3% female; FH+2 n = 40, FH+1 n = 51, FH- n = 40). Reward anticipation was measured by the monetary incentive delay task. Regression models were used to assess associations between FH status and the anticipation of large rewards in right and left NAcc regions of interest. In both studies, FH+2 youth showed blunted anticipatory reward responding in the right NAcc compared to FH+1 youth. In the MLS, FH+2 youth also had blunted anticipatory reward responding in the right NAcc compared to the FH- group. Convergent results across two separate samples provide insights into a unique vulnerability of FH+2 youth and suggest that binary FH+ versus FH- categorizations may obscure important differences within FH+ youth.

## 1. Introduction

Approximately 7.5 million youth aged 17 and younger in the U.S. live with a parent with alcohol use disorder (AUD) [1]. Through an interplay between genetic liability and temperamental and environmental factors, youth with a family history of AUD (FH+) are at elevated risk for experiencing alcohol use problems themselves [2,3,4]. For example, FH+ children were found to be three times more likely to have started drinking alcohol by age 14 and four times more likely to have been drunk by age 17 compared to children without parental AUD (FH-) [5].

The majority of studies focused on family history have categorized participants in binary terms of FH+ or FH-. Doing so, however, may ignore heterogeneity among FH+ youth. Prior studies have documented different trajectories of substance use outcomes among FH+ youth [6,7]. For example, some FH+ youth show early onset and sustained heavy use into early adulthood, while others begin using substances later in development. These findings indicate varying levels of vulnerability among FH+ youth. One potential source of this difference in vulnerability is having two biological parents with histories of AUD as opposed to one. A recent study examining AUD risk trajectories in youth from ages 14 to 30 found a positive linear association between the number of parents with AUD and offspring AUD [8]. Similarly, in a study of 12- to 15-year-olds, youth with both a father and mother who engaged in heavy episodic drinking were at heightened risk for earlier and heavier drinking compared to youth whose parents were not heavy drinkers [9].

In an attempt to understand the mechanisms underlying familial vulnerability to AUD, a number of studies have investigated reward-related brain functioning [10,11,12,13,14]. The monetary incentive delay (MID) task is often used to measure the anticipation and receipt of rewards and losses [15], with the nucleus accumbens (NAcc) showing robust activation during the anticipation of rewards [16,17]. The NAcc is within the ventral striatum and plays a central role in the brain’s dopamine system [18]. However, previous neuroimaging studies using the MID task to examine associations between reward responding and FH of substance use have yielded inconsistent results. Kwarteng et al. [11] found enhanced anticipatory reward activation in the right NAcc, whereas other studies have found no differences in anticipatory reward activation between FH+ and FH- youth [14,19,20]. The findings from Andrews et al. [21] showed that FH+ adults with a father with AUD and at least one other first- or second-degree relative with AUD had blunted NAcc activation during reward anticipation examined through an MID task. Other work has shown less anticipatory reward activation in the right NAcc in FH+ young adults compared to controls [12]. These contradictory findings may be attributable, in part, to heterogeneity within FH+ youth (e.g., having two biological parents with alcohol problems (FH+2) or one biological parent with alcohol problems (FH+1)). Given that a greater density of familial alcohol use problems is associated with a heightened vulnerability for multiple adverse outcomes in FH+ youth [22], research is needed that examines reward responding among FH+2 youth, FH+1 youth, and FH- youth.

Thus, the goal of the present study was to examine brain function in the NAcc during monetary reward anticipation measured by the MID task in FH+2, FH+1, and FH- youth from two separate studies that included similarly aged participants: (1) The Adolescent Brain Cognitive Development (ABCD) Study^®^, which is a large, diverse, nationwide study of U.S. youth; and (2) The Michigan Longitudinal Study (MLS), a community-recruited study of families from neighborhoods with high rates of alcohol problems/disorder among the parents, which yielded approximately 75% FH+ youth. The large sample size of the ABCD Study^®^ and the study design of MLS (i.e., enriched for parental AUD) allowed us to categorize the FH+ groups as FH+2 or FH+1 and test if findings would be replicated across studies. We hypothesized that both FH+2 and FH+1 groups would show aberrant NAcc activation compared to the FH- group and that the FH+2 group would show the greatest differences in NAcc activation compared to the FH- group and to a lesser extent, the FH+1 group.

## 2. Materials and Methods

### 2.1. ABCD Study^®^ Participants

The participants were 9- to 10-year-old youth (M = 9.93 years; 48% female) from the baseline sample of the ABCD Study^®^ (release 2.0). The ABCD Study^®^ recruited 11,874 children across 21 U.S. study sites. Additional details on the recruitment sites and general project information are available elsewhere [23,24]. Participants and their parent or legal guardian provided assent and informed consent, respectively, and all study procedures were approved by a central institutional review board.

### 2.2. ABCD Study^®^ Family History Measures

Parents/guardians reported on alcohol- and drug-related problems for all first- and second-degree biological relatives of the target child. The history of any alcohol-related problems in the child’s biological mother and/or father were used for the FH measure. The response options for this measure were coded as 0 = neither parent had a history of alcohol use problems (FH- n = 9690), 1 = one parent had a history of alcohol use problems (FH+1 n = 1447), or 2 = both parents had a history of alcohol use problems (FH+2 n = 223).

### 2.3. ABCD Study^®^ MID Task

The MID task [15,25] comprised three trial types where participants could win a large (USD 5) or small (USD 0.20) reward (win trial), lose a large (−USD 5) or small (−USD 0.20) reward (loss trial), or neither win nor lose money (neutral trial). The cue presentation occurred for 2000 ms and was followed by a jittered anticipation event of 1500–4000 ms, a response target presentation for 150–500 ms, and then a feedback message for 2000 ms minus the target duration. If participants pressed a response button when the response target was on the screen, they either won money (win trial), avoided losing money (loss trial), or neither won nor lost money (neutral trial); if they were too fast or too slow, they did not win money (win trial), lost money (loss trial), or neither won nor lost money (neutral trial). A practice session just prior to scanning was used to calibrate the starting response target duration so that each participant would reach a 60% accuracy rate. Additional ABCD Study^®^ MID task details are described in Casey et al. [25].

### 2.4. ABCD Study^®^ Image Data Acquisition and Processing

Centralized preprocessing and data analysis of magnetic resonance imaging (MRI) data were conducted at the ABCD Study^®^ Data Analysis and Informatics Center (DAIC). All study sites uploaded scan data to a shared server at the DAIC to maintain methodological consistency and quality control. Only NAcc regions of interest (ROIs) from scan data meeting all quality control checks by the DAIC were included in the present study. Detailed functional MRI acquisition and scanning information are provided elsewhere [25].

### 2.5. MLS Participants

The participants were 112 seven- to fifteen-year-old youth (M = 10.54 years, 39.3% female) from the MLS. Families were excluded if the child displayed signs of fetal alcohol syndrome or the child’s mother reported drinking during pregnancy. Full details on the study protocol and data collection can be found elsewhere [26]. The study protocol was approved by the University of Michigan Institutional Review Board. Participants gave signed assent, and at least one parent provided written informed consent for their child to participate.

### 2.6. MLS Family History Measures

To determine the presence of AUD, clinical assessments (Diagnostic Interview Schedule—Version 4) were conducted at the time of recruitment and then over the course of the study. Here, having a parental history of alcohol use problems was coded as 0 = neither parent had a history of alcohol use problems (FH- n = 21), 1 = one parent had a history of alcohol use problems (FH+1 n = 51), and 2 = both parents had a history of alcohol use problems (FH+2 n = 40).

### 2.7. MLS MID Task

As in the ABCD Study^®^, anticipatory reward responding was probed using the MID task [15]. The trial types and incentive amounts were identical in both studies. The reaction times to response targets were calculated based on a practice session completed just prior to scanning to achieve a success rate of approximately 60%; however, unlike the ABCD Study^®^, there was no in-task adaptive calibration. Additional MLS MID task details are described elsewhere [12,27,28].

### 2.8. MLS Image Data Acquisition and Processing

Information on image data acquisition and processing is provided in the Supplemental Materials.

### 2.9. Analytic Strategy

In the ABCD Study^®^, FH group differences in brain activation in the left and right NAcc during the anticipation of large rewards vs. neutral (i.e., “Win USD 5” vs. “No money at stake”) were examined using mixed effects linear regression in Stata (v15.1). Six separate mixed effects linear regression models were computed. The mean beta weights from the left or right NAcc ROIs were the outcome measures, and the FH group contrast (FH- vs. FH+1, FH- vs. FH+2, or FH+1 vs. FH+2) was the fixed-effect predictor. The random effects in each model were site and family identifiers, the latter accounting for the possible impact of sibling correlations.

In the MLS, FH group differences in brain activation in the left and right NAcc during the anticipation of large rewards vs. neutral (i.e., “Win USD 5” vs. “No money at stake”) were examined using linear regression in SPSS (v27). Six separate linear regression models were computed: Beta weights from the left or right NAcc ROIs were the outcome measures, and FH group contrasts (FH- vs. FH+1, FH- vs. FH+2, or FH+1 vs. FH+2) were modeled separately as predictors.

## 3. Results

### 3.1. ABCD Study^®^ Results

**Figure 1:**
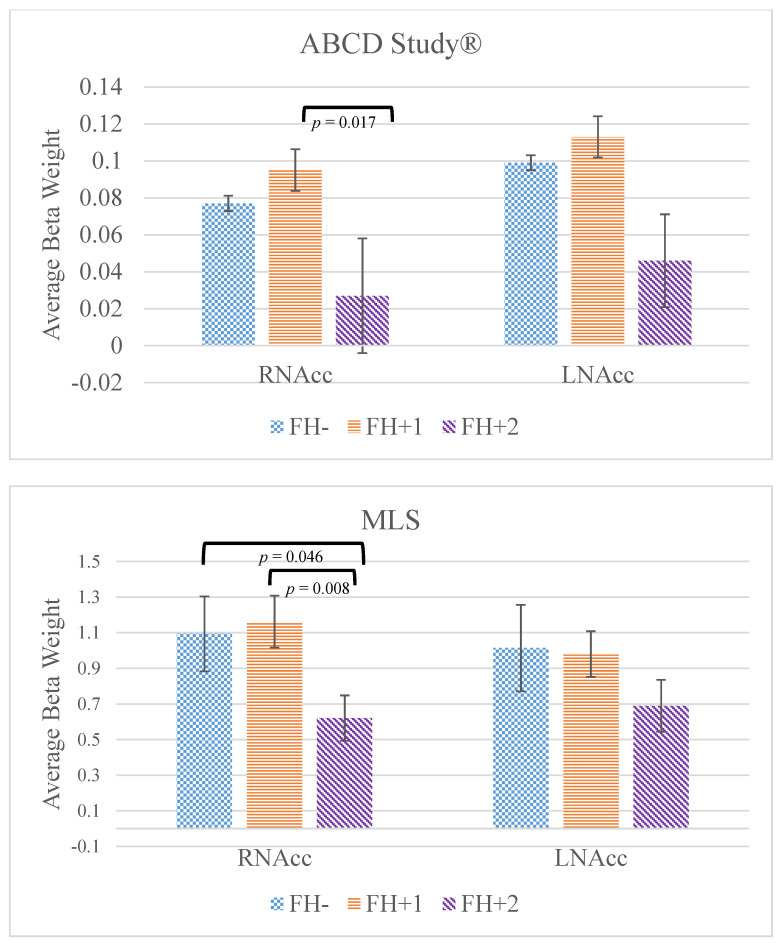
Right and left nucleus accumbens (NAcc) activation during the anticipation of a large rewards versus neutral contrast in the ABCD Study^®^ (**top**) and MLS (**bottom**). Error bars denote standard error.

As shown in Figure 1 and Table 1, the FH+2 youth had significantly lower right NAcc activation associated with a large reward versus neutral anticipation compared to the FH+1 youth. The overall linear mixed regression model for large reward anticipation in the right NAcc was significant (Wald χ^2^ = 6.51, *p* = 0.039). Although FH+2 showed lower anticipatory reward activation in the left NAcc compared to both FH+1 and FH- youth, the overall model was not significant (Wald χ^2^ = 5.67, *p* = 0.059).

### 3.2. MLS Results

As shown in Figure 1 and Table 1, the FH+2 youth had significantly lower right NAcc activation associated with a large reward versus neutral anticipation compared to the FH+1 youth and FH- youth. The overall linear regression model for large reward anticipation in the right NAcc was significant (*F*(1108) = 4.972, *p* = 0.028). Although there were significant group differences for the left NAcc model, the model fit statistics indicated that this model was not significant (*F*(1108) = 2.076, *p* = 0.152).

Exploratory regression analyses were used to examine if the measure of any FH versus no parental history of alcohol use problems, rather than the three-category FH measure, was associated with significant differences in NAcc activation. We conducted these analyses to determine if the binary FH- vs. FH+ group classification may have obscured differences between groups classified as FH-, FH+1, and FH+2. In the ABCD Study^®^ sample, FH+ vs. FH- was not a significant predictor of a large reward versus neutral anticipation in the right NAcc model (β = 0.01, *p* = 0.476) or the left NAcc model (β = 0.001, *p* = 0.891). In the MLS sample, FH+ vs. FH- was not a significant predictor of a large reward versus neutral anticipation in the right NAcc model (β = −0.33, *p* = 0.198) or the left NAcc model (β = −0.28, *p* = 0.278).

## 4. Discussion

The present study examined anticipatory reward activation in the NAcc among youth with both, one, or no biological parents with alcohol use problems in two independent samples: a large, national study and a community-based study enriched for parental AUD. The study hypotheses were partially supported. In both studies, the FH+2 group had significantly lower anticipatory reward activation in the right NAcc compared to the FH+1 group; in the MLS sample, FH+2 also had lower right NAcc activation compared to FH- youth. There were no significant differences between the FH+1 and FH- youth, however. The findings suggest that a blunted NAcc response to anticipatory rewards may be an indicator of reward dysfunction unique to FH+2 youth.

Our findings align, to a certain extent, with the prior literature showing differences between FH+ and FH- individuals specifically in the right NAcc [11,12]. Unlike the present study, Kwarteng et al. [11] found that FH+ youth had significantly greater activation in the right NAcc during anticipatory reward responding compared to FH- youth. This discrepancy may be due to differences in how FH+ groups were classified by Kwarteng et al. [11] (e.g., binary FH+ versus FH- groups, FH+ youth having biological parents with at least two problems with alcohol). As in the present study, but in an older sample of young adults from the MLS, Yau et al. [12] found decreased activation in the right NAcc in FH+ youth compared to FH- controls. Interestingly, these results were unique to FH+ youth who were low drinkers, had never binged, and reported few alcohol-related problems, suggesting that lower anticipatory reward activation in the right NAcc may be observed in FH+ youth with low levels of prior and current alcohol consumption. It will be important for future studies to assess potential differences in NAcc activation among FH+ youth longitudinally, both prior to and after the onset of substance use.

Considering that FH+2 youth may experience greater vulnerability across many domains of functioning and psychopathologies [8,29], the results from the present study may be capturing reward hypoactivation as a broad liability marker for psychopathology. Indeed, reward dysfunction has been identified as a transdiagnostic marker of psychopathology [30]. Blunted activity in the ventral striatum, which contains the NAcc, is associated with disorders involving negative mood states, including substance use as well as schizophrenia and depression [31]. In recent work using data from the baseline sample of the ABCD Study^®^, having a higher family loading of substance use problems was associated with a higher general psychopathology (p-factor) [32]. Future work is needed to better understand the interplay between psychopathology and social disorganization in FH+2 youth in relation to reward responding.

A key strength of the present study is its use of both the ABCD Study^®^ and MLS samples, providing support for the external validity and reliability of our results. The large sample size of the ABCD Study^®^ and the study design of the MLS allowed for differentiating between FH+2 and FH+1 youth. Non-significant results when combining FH+2 and FH+1 into a single FH+ group compared to FH- youth suggest that single categorizations of FH+ individuals may obscure within-group heterogeneity. Some limitations should also be noted. Although the mean ages were comparable across the study samples, there was also a wider age range in the MLS sample. It is possible that age-related changes in dopamine-driven reward motivation may have impacted our results [33]. Second, we did not differentiate between youth whose biological parent with alcohol problems did or did not live with them, and we were not able to disentangle environmental- and genetic-related risk. Finally, while comparable in relation to the trial and cue types, the MID tasks used in the ABCD Study^®^ and the MLS were not identical (e.g., the jittered anticipation and adaptive success rate in the ABCD Study^®^). However, the same phenomena of interest were assessed in both the ABCD Study^®^ and MLS.

## 5. Conclusions

In sum, accounting for the nuance in FH status showed that having both biological parents with alcohol use problems was associated with blunted activation in the right NAcc during reward anticipation. The results were convergent across both the ABCD Study^®^ and MLS samples. It will be important for future work to examine the extent to which anticipatory reward responding in the NAcc and other regions predicts substance use initiation and trajectories among FH+2, FH+1, and FH- youth. Early indicators of risk density may provide information on differences in neural function, including reward responding, as prevention targets.

## Figures and Tables

**Table 1:** Regression model results by ABCD Study^®^ and MLS family history groups.

	ABCD Study^®^		
**Right NAcc**	Beta Coefficient	95% Confidence Interval	*p*-value
FH- vs. FH+1	0.019	−0.004, 0.042	0.100
FH- vs. FH+2	−0.053	−0.108, 0.003	0.064
FH+2 vs. FH+1	−0.072	−0.131, −0.013	0.017
	**MLS**		
**Right NAcc**	Beta Coefficient	95% Confidence Interval	*p*-value
FH- vs. FH+1	0.068	−0.457, 0.594	0.797
FH- vs. FH+2	−0.236	−0.468, −0.004	0.046
FH+2 vs. FH+1	−0.541	−0.939, −0.143	0.008
	**ABCD Study^®^**		
**Left NAcc**	Beta Coefficient	95% Confidence Interval	*p*-value
FH- vs. FH+1	0.014	−0.009, 0.036	0.237
FH- vs. FH+2	−0.055	−0.109, −0.001	0.046
FH+2 vs. FH+1	−0.069	−0.126, −0.011	0.019
	**MLS**		
**Left NAcc**	Beta Coefficient	95% Confidence Interval	*p*-value
FH- vs. FH+1	−0.034	−0.536, 0.468	0.892
FH- vs. FH+2	−0.162	−0.429, 0.105	0.229
FH+2 vs. FH+1	−0.290	−0.676, 0.095	0.138

Note. ABCD = Adolescent Brain Cognitive Development; MLS = Michigan Longitudinal Study; NAcc = nucleus accumbens; FH- = no parental history of alcohol use problems; FH+1 = one biological parent with alcohol use problems; FH+2 = two biological parents with alcohol use problems; the contrast of interest for both the right NAcc and left NAcc was anticipation of a large reward versus neutral trials.

## Data Availability

ABCD Study^®^: Only researchers with an approved NDA Data Use Certification may obtain ABCD Study^®^ data. MLS: Not applicable.
